# RAFT Polymerization of *Tert*-Butyldimethylsilyl Methacrylate: Kinetic Study and Determination of Rate Coefficients

**DOI:** 10.3390/polym10020224

**Published:** 2018-02-24

**Authors:** Minh Ngoc Nguyen, André Margaillan, Quang Trung Pham, Christine Bressy

**Affiliations:** 1 Faculty of Chemistry, VNU University of Science, Hanoi, 19 Le Thanh Tong, Hoan Kiem, Hanoi 100000, Vietnam; trungpham781@hus.edu.vn; 2 Laboratoire MAtériaux Polymères-Interfaces-Environment Marin (EA 4323 MAPIEM), Université de Toulon, CS 60584-83041 Toulon CEDEX 9, France; andre.margaillan@univ-tln.fr

**Keywords:** reversible addition-fragmentation chain transfer (RAFT), trialkylsilyl methacrylate, rate coefficients, simulation, PREDICI^®^

## Abstract

Well-defined poly(*tert*-butyldimethylsilyl methacrylate)s (TBDMSMA) were prepared by the reversible addition-fragmentation chain transfer (RAFT) process using cyanoisopropyl dithiobenzoate (CPDB) as chain-transfer agents (CTA). The experimentally obtained molecular weight distributions are narrow and shift linearly with monomer conversion. Propagation rate coefficients (*k*_p_) and termination rate coefficients (*k*_t_) for free radical polymerization of TBDMSMA have been determined for a range of temperature between 50 and 80 °C using the pulsed laser polymerization-size-exclusion chromatography (PLP-SEC) method and the kinetic method via steady-state rate measurement, respectively. The CPDB-mediated RAFT polymerization of TBDMSMA has been subjected to a combined experimental and PREDICI modeling study at 70 °C. The rate coefficient for the addition reaction to RAFT agent (*k*_β1_, *k*_β2_) and to polymeric RAFT agent (*k*_β_) is estimated to be approximately 1.8 × 10^4^ L·mol^−1^·s^−1^ and for the fragmentation reaction of intermediate RAFT radicals in the pre-equilibrium (*k*_-β1_, *k*_-β2_) and main equilibrium (*k*_-β_) is close to 2.0 × 10^−2^ s^−1^. The transfer rate coefficient (*k*_tr_) to cyanoisopropyl dithiobenzoate is found to be close to 9.0 × 10^3^ L·mol^−1^·s^−1^ and the chain-transfer constant (*C*_tr_) for CPDB-mediated RAFT polymerization of TBDMSMA is about 9.3.

## 1. Introduction

Polymers containing hydrolysable bonds have been developed for many years in pharmaceutical, biomedical, and antifouling areas. Their chemical structures are designed to release drugs or biocides through the erosion of polymer [1,2]. In our research group, polymers containing trialkylsilyl esters as pendant groups have been developed as polymeric binders for self-polishing antifouling coatings [3,4]. These silyl ester derivatives are readily subjected to alkaline hydrolysis in seawater yielding to a remaining acid-functional polymer, which becomes seawater-soluble and gradually swept from the surface of the coating. Therefore, this chemically controlled erosion of the coating can be used in processes based on the leaching of active compounds such as biocides in antifouling paints. Polymeric resins bearing hydrolysable pendant groups have been prepared through chemical modification or polymerization methods [5].

**Scheme 1:**
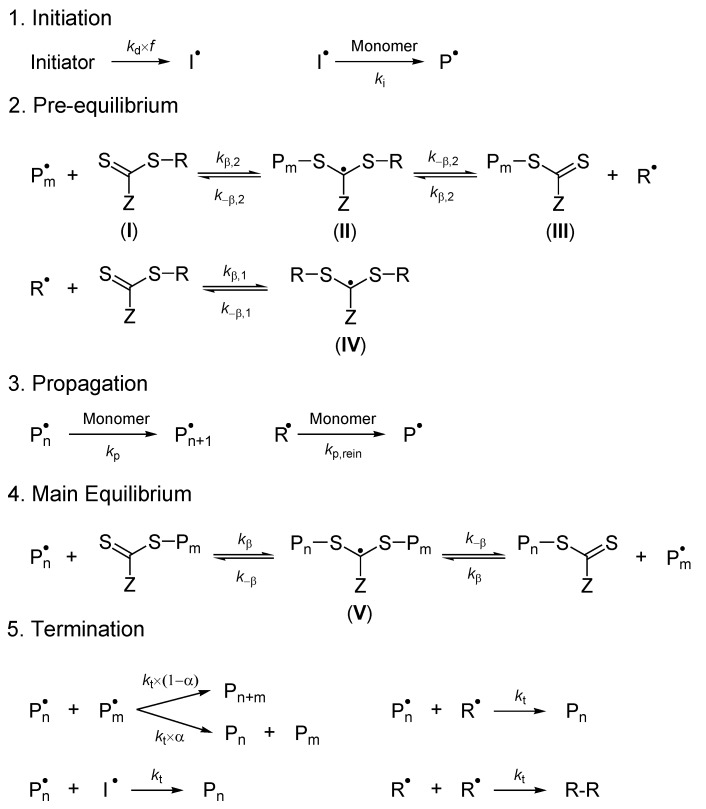
Detailed mechanism of the RAFT process.

Among the reversible deactivation radical polymerization (RDRP) methods, the reversible addition-fragmentation chain transfer (RAFT) polymerization has been revealed as a robust method to prepare homopolymers and complex macromolecular architectures such as block, comb, and star copolymers with controlled molecular weights and low dispersity [6,7]. The success of the RAFT process is based on the use of efficient thiocarbonylthio compounds as reversible chain-transfer agents (CTA) which ensure the control of the polymerization by the establishment of a dynamic equilibrium between active propagating macroradicals and dormant polymer chains. The effectiveness of CTAs in terms of transfer ability, control of the molecular weight, and the dispersity depends strongly on the nature of the Z and R groups (see Scheme 1) [6]. Highly effective chain-transfer agents are thiocarbonylthio compounds, where R is a free radical leaving group which is able to reinitiate the monomer polymerization and Z is a group that modifies both the reactivity of carbon sulphur double bond and the stability of the intermediate macroradical. In an ideal system, the chain transfer and equilibrium steps should be fast to limit the termination events without influencing the rate of polymerization. Nevertheless, retardation effects as well as the presence of an inhibition period have been reported to occur in many dithiobenzoate-mediated RAFT polymerizations, including methacrylate monomers [8,9,10,11]. These two kinetic effects have been reviewed in 2006 by an International Union of Pure and Applied Chemistry (IUPAC) task group [12] and recently updated by G. Moad [13].

In our previous papers [4,5,14], the RAFT polymerization and copolymerization of *tert*-butyldimethylsilyl methacrylate (TBDMSMA), an interesting precursor for hydrosoluble film-forming polymers, have been studied. The controlled characters of the TBDMSMA polymerization using 2-phenylprop-2-yl dithiobenzoate (i.e., cumyl dithiobenzoate, CDB) or 2-cyanoprop-2-yl dithiobenzoate (i.e., cyanoisopropyl dithiobenzoate, CPDB) as RAFT agent have been demonstrated. However, a short period of inhibition and a molecular weight extrapolated at zero conversion different from zero have been observed. Some authors [8,15] suggested that the pre-equilibrium can have a significant effect on the polymerization kinetics, especially on the effects associated with inhibition. This article attempts to obtain more information about the kinetics of the CPDB-mediated RAFT polymerization of TBDMSMA, through the combination of both experimental and theoretical approaches. The propagation rate coefficient (*k*_p_) for TBDMSMA free-radical polymerization was determined by pulsed-laser polymerization (PLP) in conjunction with the analysis of the molecular weight distribution (MWD) via size-exclusion chromatography (SEC). Determination of *k*_t_ was carried out using kinetic method and via steady-state rate measurement. Modeling of the time evolution of molecular weight, monomer conversion, RAFT agent conversion, and dispersity at various initial RAFT agent concentrations using PREDICI^®^ software (Dr. M. Wulkow Computing in Technology, Rastede, Germany) was comprehensively studied and compared to experimental data. The aim is to evaluate the rate coefficients, especially those associated with the addition-fragmentation equilibrium and to give a better understanding of the mechanism and the kinetics of the RAFT polymerization of TBDMSMA.

## 2. Experimental

### 2.1. Materials 

All chemicals were purchased from Acros or Aldrich. Toluene was distilled over the blue benzophenone–Na complex prior to use. 2,2’-Azobis(isobutyronitrile) (AIBN) was purified by recrystallization from methanol. 2-cyanoprop-2-yl dithiobenzoate (CPDB) with a purity superior to 98% were used as received without further purification. *Tert*-butyldimethylsilyl methacrylate (TBDMSMA) was prepared according to the literature [14].

### 2.2. Polymerization Procedures 

A mixture of TBDMSMA, CTA, and AIBN in toluene was introduced in a 100 mL, three-neck round bottom flask. The flask was degassed by three freeze–pump–thaw cycles, filled with argon and sealed with a rubber septum in Teflon. The reaction flask was then placed in a thermostatic oil bath at a set temperature. For kinetic study, samples were withdrawn through a degassed syringe at timed intervals for ^1^H NMR and SEC analyses. Recipes of polymerizations are summarized in Table 1. For the determination of termination rate coefficients for free-radical polymerization of TBDMSMA (entries 1–3), monomer conversion was limited to 10–15%.

### 2.3. PLP Investigations 

TBDMSMA, toluene, and the photoinitiator Darocur 1173 were mixed, poured into a double-walled cylindrical cuvette (Starna, 65.14/Q/10, Spectrosil-fused quartz, path length: 10 mm, Starna, Atascadero, CA, USA), and degassed with argon for 5 min. Temperature was controlled with a heat-transfer fluid using a thermostat. After thermostating for 15 min, the PLP experiment was performed with an ATLEX-I laser (ATL Lasertechnik GmbH, pulse width: 20 nm, maximum pulse energy: 7 mJ, maximum pulse repetition rate: 1000 Hz, ATL Lasertechnik, Wermelskirchen, Germany) operating on the XeF line at 351 nm. Pulsed laser polymerizations were performed at repetition rates between 1 and 100 Hz. After laser irradiation, the polymer/monomer mixture was poured into a flask containing hydroquinone inhibitor. Residual monomer was removed by evaporation under reduced pressure. Conditions and results for this study are summarized in Table 2.

### 2.4. Characterization Methods

The monomer and the chain-transfer agent conversions were determined by proton nuclear magnetic resonance (^1^H NMR) spectroscopy. ^1^H NMR spectra were recorded on a Bruker Avance (400 MHz) spectrometer with CDCl_3_ or toluene-d_8_ as solvent. ^1^H NMR spectra of TBDMSMA and PTBDMSMA are presented in Figure S1 (in Supplementary Materials). Monomer conversion (p) was determined using the integral of two protons (I_a_) of double bonds (at 5.58 ppm and 6.10 ppm) of the monomer and the integral of six protons of the remaining monomer (I_b_ at 0.30 ppm) and of the resulting polymer (I_b’_ at 0.22 ppm).

$$p=\frac{I_{b}+I_{b’}-3I_{a}}{I_{b}+I_{b’}}$$

The apparent average molecular weights (*M*_n_ and *M*_w_) and the molecular weight distribution or dispersity (*M*_w_/*M*_n_ or *Đ*) of polymers were determined by size exclusion chromatography (SEC) on a Waters 501 pump equipped with a refractive-index detector (DRI 410Waters, Milford, MA, USA), a Kontron 432 HPLC UV detector (Kontron Instrument, Zurich, Switzerland), and five Waters Styragel HR columns (2 HR0.5, HR1, HR3, and HR4; 7.8 × 300 mm). Tetrahydrofuran was used as an eluent at 30 °C and at a flow rate of 1.0 mL·min^−1^. The apparent average molecular weight and dispersity data were compared against narrow standards of poly(methyl methacrylate) (PMMA; *M*_p_ = 620 to 3.64 × 10^5^ g·mol^−1^) obtained from Polymer Laboratories. The corrected molecular weight values were obtained via the reported Mark–Houwink–Sakurada parameters for PTBDMSMA [14]. The SEC chromatograms of resulting polymers (entries 4–9 in Table 1) are presented in Figure S2 (in Supplementary Materials).

### 2.5. Simulation 

The PREDICI^®^ model for the CPDB-mediated polymerization of TBDMSMA was constructed on the basis of kinetic and thermodynamic parameters from literature. The kinetic steps for RAFT polymerization were shown in Scheme 1. They were implemented into the PREDICI^®^ program package using the kinetic parameters, the initial values of reactant concentrations, and the individual reaction steps. The program package uses a method of approximation for countable differential equation systems and a special time discretization, which is especially efficient in the context of polymerization reactions [14]. For the CPDB-mediated TBDMSMA polymerization, the kinetic scheme consists of the initiation, a pre-equilibrium, propagation and re-initiation, the main or core equilibrium, and termination processes.

Scheme S1 (in Supplementary Materials) describes the translation of the chemical model given in Scheme 1 into a form that can be implemented into the PREDICI^®^ program package. For the main equilibrium, two fictive species Q_1_ and Q_2_ (each acting as a chain-length memory for the macroradical RAFT species) are introduced to overcome the difficulty to assign two different chain lengths to one radical species. Further detailed discussions about the implement of the RAFT process in PREDICI software can be found in the literature [15,16,17,18]. 

## 3. Results and Discussion

### 3.1. Determination of k_p_ and k_t_

**Figure 1:**
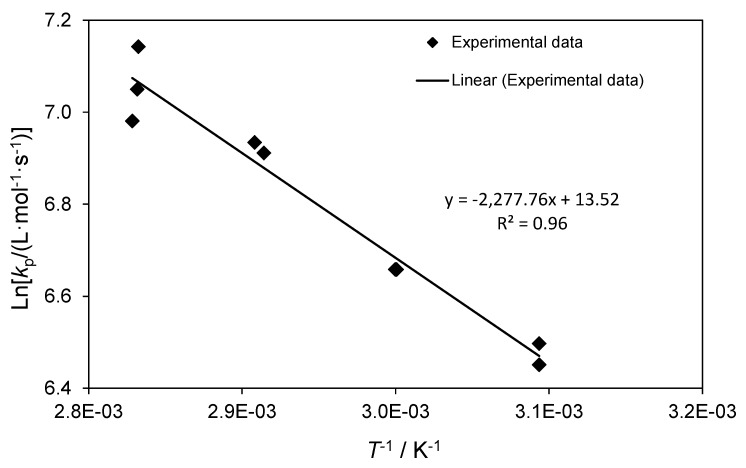
Arrhenius plot of the propagation rate coefficient *k*_p_ for *tert*-butyldimethylsilyl methacrylate polymerization. Points: experimental data; line: best fit of linearized Arrhenius equation to points.

The propagation rate coefficient (*k*_p_) for TBDMSMA free-radical polymerization was determined by pulsed-laser polymerization (PLP) in conjunction with the analysis of the molecular weight distribution (MWD) via size-exclusion chromatography (SEC) as recommended by the IUPAC Working Party on *Modeling of Polymerization Kinetics and Processes* [19,20]. PLP-SEC investigations of free-radical polymerization of TBDMSMA in toluene were performed at low conversions (<1.5%) and atmospheric pressure for different temperatures ranging from 50 to 80 °C. The obtained values of *k*_p_ (Table 2) are presented graphically in Figure 1 as ln*k*_p_ versus 1/*T*, where *T* is temperature in K. The linear regression line represents the best fit of the experimental data points and can be expressed by the Arrhenius equation (Equation (1)) as

(1) $$\ln\left[k_{p}{/(L\;\mathrm{mol}}^{-1}{\;s}^{-1})\right]=\ln\left[10^{5.87}\right]-\frac{18.94{\mathrm{kJ}\;\mathrm{mol}}^{-1}}{R\cdot T}$$

The *k*_p_ values of TBDMSMA free-radical polymerization in the range of temperature between 50 and 80 °C are comparable with those found for the free-radical polymerization of methacrylate monomers [19,20,21].

The determination of *k*_t_ was carried out using kinetic method and via steady-state rate measurement [22,23]. In free radical polymerization, the rate of propagation, and therefore the rate of polymerization $R_{p}$ can be expressed by Equation (2) according to the steady-state assumption.

(2) $$R_{p}=-\frac{d[M]}{dt}=k_{p}[M]{\left(\frac{R_{i}}{{2k}_{t}}\right)}^{1/2}=k_{p}[M]{\left(\frac{{fk}_{d}[I]}{k_{t}}\right)}^{1/2}$$

where [M] and [I] are the concentrations of the monomer and the initiator, respectively, *R*_i_ is the initiation rate, *k*_d_ is the decomposition rate coefficient, *f* is the initiator efficiency and *k*_t_ is the termination rate coefficient. For the present study, the values of initiator decomposition rate coefficient (*k*_d_) and initiator efficiency (*f*) for AIBN decomposition at three different temperatures were taken from literature [21].

In order to take into account the effect on rate of diminishing monomer concentration with time, the following arithmetic rearranged from of Equation (2) is used.

(3) $$\ln({\left[M\right]}_{0}/\left[M]\right)/{\left[I\right]}^{1/2}=\ln(1/(1-x))/{\left[I\right]}^{1/2}=k_{p}{\left(\frac{fk_{d}}{k_{t}}\right)}^{1/2}\times t$$

**Figure 2:**
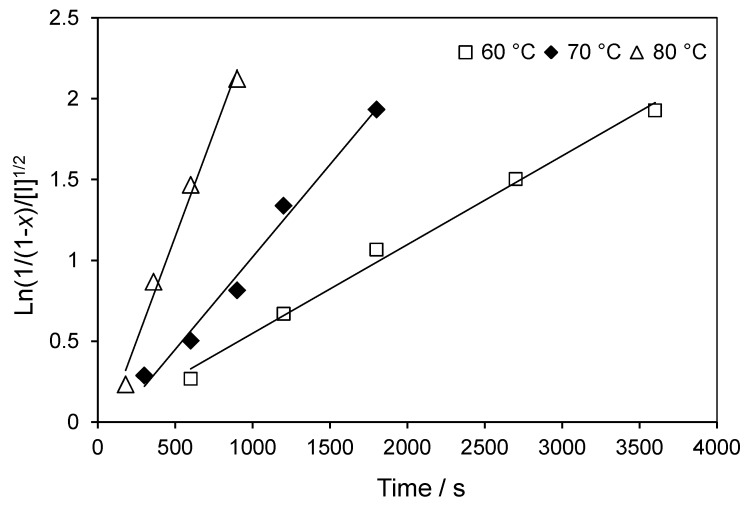
Plot of ln(1/(1 − *x*))/[I]^1/2^ versus time for free-radical polymerization of TBDMSMA at 60, 70, and 80 °C in toluene. [TBDMSMA] = 1.5 mol·L^−1^, [AIBN] = 6.0 × 10^−3^ mol·L^−1^.

Here *x* is the fractional conversion of monomer into polymer, *t* the reaction time. In this study, the monomer conversion was limited to up to 15%. Variation with reaction time of ln(1/(1 − *x*)) at different temperatures is shown in Figure 2. It is clear that ln(1/(1 − *x*))/[I]^1/2^ increases linearly with reaction time within experimental error. Therefore, *k*_t_ of the free radical polymerization of TBDMSMA for different temperatures could be assessed from the slope of ln(1/(1 − *x*))/[I]^1/2^ versus reaction time (Table 3). *k*_t_ values ranging from 1.49 × 10^7^ L·mol^−1^·s^−1^ at 60 °C to 1.90 × 10^7^ L·mol^−1^·s^−1^ at 80 °C are of the same order of magnitude as those found for free-radical polymerization of methacrylate monomers [21,22,23,24].

### 3.2. Validation of the PREDICI^®^ Model and Assessment of the Rate Coefficients via Simulations

To validate the model and to assess the unknown rate coefficients (*k*_β_,_i_ and *k-*_β,i_ with i = 0, 1, 2), the kinetic scheme given in Scheme 1 was fitted to the experimental data using the PREDICI^®^ simulation package. We used four sets of experimental data for the cyanoisopropyl dithiobenzoate-mediated TBDMSMA polymerizations in toluene at 70 °C, each set of experimental data representing a specific concentration of the RAFT agent. The resulting information includes *M*_w_, dispersity (*Đ*), monomer conversion, and RAFT agent conversion at each reaction time. For modeling the time-dependent evolution of these experimental features using PREDICI^®^, we used a fixed set of kinetic parameters including the effective initiator decomposition rate coefficient (*k*_d,eff_ = *k*_d_ × *f*), the primary radical and long-chain propagation coefficients (*k*_i_, *k*_p_), the re-initiation rate coefficient (*k*_p, rein_), and the termination rate coefficient (*k*_t_). The reaction of an initiator derived radical with monomer is assumed to be faster (about five times) than the long-chain propagation rate coefficient as a chain-length dependence of the propagation rate coefficient has been mentioned for MMA and styrene polymerizations [18,25]. The re-initiation rate coefficient (*k*_p, rein_) is assumed to be equal to *k*_i_ because the cyanoisopropyl radical, R^•^, formed from the fragmentation of the macroRAFT radical (specie II, Scheme 1) has the same chemical structure compared to the radical I^•^ formed by the decomposition of AIBN. Because of the strong steric hindrance of *tert*-butyldimethylsilyl methacrylate group, it is assumed that bimolecular termination between two macroradicals (step 5, Scheme 1) could not take place by combination. Therefore, α was set equal to 1 for all simulations. The values of fixed parameters are summarized in Table 4.

It is also important to choose the appropriate starting values of unknown parameters for the simulations. For the RAFT process to be efficient, some points must be considered such as: (i) the addition of a macroradical to a polymeric RAFT species is considered to proceed rather fast, comparable to the rate coefficient of propagation and (ii) the fragmentation reaction, being a unimolecular reaction, should have a rather low rate coefficient [17,18]. The same value for all *k*_β,i_ and for all *k-*_β,i_ was used as starting input values. Based on these arguments, the starting values for *k*_β,i_ and *k-*_β,i_ were set to 10^5^ L·mol^−1^·s^−1^, and 10^−2^ s^−1^, respectively.

In the next step, these starting parameters were used for modeling the time-dependent evolution of the experimental features such as *M*_w_, dispersity (*Đ*), monomer conversion, and RAFT agent conversion. The careful comparison of experimental and simulated data led to additional improvements and refinements in the magnitude of the rate coefficients. The final set of rate coefficients given in Table 5 can obtain an optimal description of the experimental data. It is worth noting that the order of magnitude for the values of *k*_β,i_ and *k-*_β,i_ are close to those of the starting values. These values of rate coefficients were used for all simulations unless otherwise indicated.

**Figure 3:**
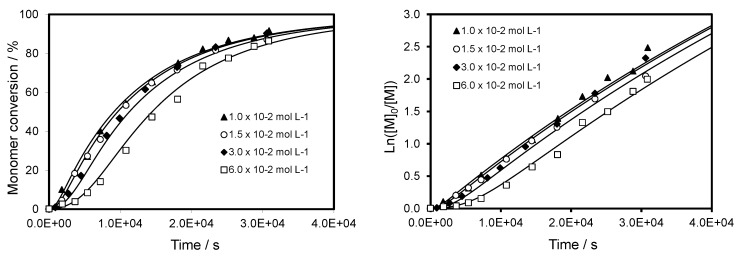
Evolution of monomer conversion and ln([M]_0_/[M]) versus reaction time for CPDB-mediated polymerization of TBDMSMA in toluene at 70 °C with initial concentration of CPDB ranging from 1.5 × 10^−2^ to 6.0 × 10^−2^ mol·L^−1^. The graph compares predicted values (lines) with experimental data (points).

Figure 3 shows the evolution of the monomer conversion and ln([M]_0_/[M]) with reaction time for different initial concentrations of cyanoisopropyl dithiobenzoate-mediated TBDMSMA polymerizations in toluene at 70 °C. The full lines given in Figure 3 represent the fitting results. It is clear that the agreement between the modeled and experimental data sets is good up to high monomer conversion. The linear variation of ln([M]_0_/[M]) versus reaction time suggests a first-order kinetics for the TBDMSMA polymerization. Additionally, the nearly identical polymerization rate is observed for all initial concentrations of RAFT agent. This means that no retardation is related to this polymerization; i.e., the polymerization rate is almost independent of the concentration of the initial RAFT agent. However, Figure 1 shows an inhibition period at the beginning of the polymerization and it becomes more pronounced when a high concentration of RAFT agent was used. It should be noted that no inhibition was observed in free-radical polymerization of TBDMSMA as shown in Figure 2. Because every RAFT polymerization is subject to an initial period of slow polymerization, which does not qualify as complete inhibition, the period of inhibition (*t*_inhib_) determined by linearly fitting the approximately linear parts of the conversion versus time profiles [8,26], varies from about 1000 s for an initial RAFT agent concentration of 1.0 × 10^−2^ mol·L^−1^ to about 5000 s for an initial RAFT agent concentration of 6.0 × 10^−2^ mol·L^−1^. In the case of the CPDB-mediated RAFT polymerization of TBDMSMA, complete inhibition is assumed to be observed at very high RAFT agent concentration. The cause of this inhibition may either be associated with the ability of the leaving group of the initial RAFT agent to reinitiate the polymerization or with the slow fragmentation of the initial pre-equilibrium RAFT radical (species II and IV, Scheme 1). In our case, the inhibition effect is attributed rather to the slow fragmentation of the initial pre-equilibrium RAFT radical than to a slow re-initiation. Actually, we mentioned above that the value of *k*_p, rein_ is equal to that of *k*_i_ because the radical involved in the re-initiation reaction is identical to the primary initiating radical.

**Figure 4:**
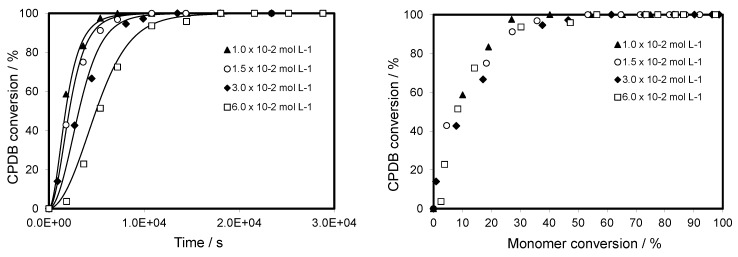
Evolution of CPDB conversion versus reaction time and of monomer conversion versus CPDB conversion for CPDB-mediated polymerization of TBDMSMA in toluene at 70 °C with initial concentration of CPDB ranging from 1.5 × 10^−2^ to 6.0 × 10^−2^ mol·L^−1^. The graph compares predicted values (lines) with experimental data (points).

To better understand the slow fragmentation of the initial pre-equilibrium RAFT radical, the conversion of the RAFT agent versus time was followed experimentally and by modeling and is graphically represented in Figure 4.

Again, the agreement between the modeled and experimental data sets is excellent. It can be clearly seen from Figure 4 that the RAFT agent is not very fast consumed. It takes about 7000 s and more than 15000 s to make it completely consumed for an initial CPDB concentration of 1.0 × 10^−2^ mol·L^−1^ and 6.0 × 10^−2^ mol·L^−1^, respectively. The evolution of the RAFT agent conversion with monomer conversion is also studied. It is interesting to note that RAFT agent is completely consumed at a monomer conversion of about 40% and the evolution of the RAFT agent conversion versus monomer conversion is similar whatever the RAFT agent concentrations. In the previous study [14], we have reported that the resulting polymers really have a dithiobenzoate end-group derived from CPDB and it is possible to evaluate the CTA efficiency from the ^1^H NMR spectrum of PTBDMSMA. This is likely related to the control of the molecular weight of the polymer that will be discussed in the following part.

**Figure 5:**
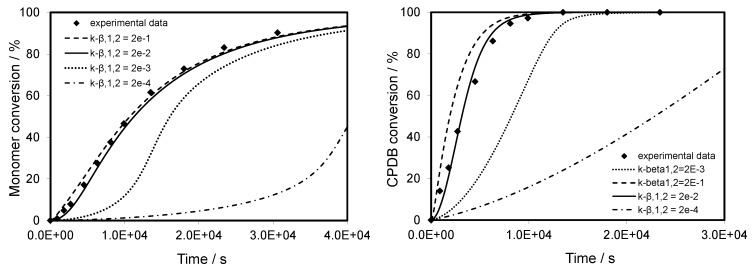
Evolution of monomer and CPDB conversion versus reaction time for CPDB-mediated polymerization of TBDMSMA in toluene at 70 °C with initial concentration of CPDB of 3.0 × 10^−2^ mol·L^−1^. The graph compares predicted values (lines) with experimental data (points).

Figure 5 shows the effects of *k-*_β,1_ and *k-*_β,2_ values (corresponding to the fragmentation of the intermediate RAFT radicals in the pre-equilibrium) on the rate of consumption of monomer and RAFT agent. It is clear that inhibition effects on monomer conversion are observed, when either *k-*_β,1_ and *k-*_β,2_ are smaller than 10^−1^ s^−1^, i.e., when they become smaller than the value of *k-*_β_ of the core equilibrium. By lowering *k-*_β,1_ and *k-*_β,2_ below a value of 10^−3^ s^−1^, an inhibition period of more than 3000 s is found. The optimal value of 2.0 × 10^−2^ s^−1^ obtained for both *k-*_β,1_ and *k-*_β,2_ makes it possible to model the experimental data perfectly. 

**Figure 6:**
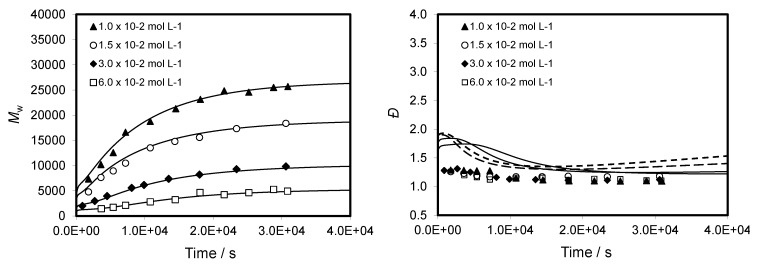
Evolution of *M*_w_ and *Đ* versus reaction time for CPDB-mediated polymerization of TBDMSMA in toluene at 70 °C with initial concentration of CPDB ranging from 1.5 × 10^−2^ to 6.0 × 10^−2^ mol·L^−1^. The graph compares predicted values (lines) with experimental data (points).

In Figure 6, the evolutions of the weight-average molecular weight (*M*_w_) and the dispersity (*Đ* or *M*_w_/*M*_n_) as a function of the reaction time were plotted for both experimental and simulated data for four initial RAFT agent concentrations. It can be clearly seen that a good agreement between experimental and simulated data was observed up to high monomer conversions. Decreases in molecular weight with increasing the initial RAFT agent concentration are in accordance with the principle of RAFT polymerization, i.e., molecular weights are predicted by the concentration ratio of monomer to RAFT agent. Additionally, the CPDB-mediated RAFT polymerizations of TBDMSMA are consistent with controlled processes, as revealed by the linear evolution of the molecular weight with monomer conversion (see Figure S3 in Supplementary Materials for details). These results are in good agreement with the above-mentioned observation in which the concentration ratio between monomer and RAFT agent ([Monomer]/[CTA]) at a given time is identical whatever the initial concentration of RAFT agent.

However, the *M*_w_-time profiles showed an *M*_w_ value at zero conversion different from zero and the higher the initial concentration ratio of monomer to RAFT agent is, the greater the deviation is. This phenomenon has been observed for various RAFT polymerizations of methacrylate monomers and has been related to a low chain-transfer constant (*C*_tr_) of the RAFT agent in the pre-equilibrium step [14,18]. In this study, the rate coefficients *k*_β,1_ and *k*_β,2_ for the addition steps in pre-equilibrium were estimated to be equal to 1.8 × 10^4^ L·mol^−1^·s^−1^. Thus, we could calculate the chain-transfer rate coefficient (*k*_tr_ = 9.0 × 10^3^ L·mol^−1^·s^−1^) and the chain-transfer constant (*C*_tr_ = 9.3) for CPDB-mediated RAFT polymerization of TBDMSMA by using Equations (4) and (5)

(4) $$k_{\mathrm{tr}}=\frac{1}{2}k_{\beta }$$

(5) $$C_{\mathrm{tr}}=\frac{k_{\mathrm{tr}}}{k_{p}}$$

The magnitude of the *C*_tr_ value perfectly reflects the non-reversible (i.e., “conventional”) transfer behavior at the beginning of the polymerization. After a sufficient amount of time, the proportion of transferred (dormant) chains is high enough so that chain transfer occurs mainly between propagating radicals and dormant chains.

Since the molecular weight distribution is controlled by the equilibrium constant of the main equilibrium (*K*_eq_ = *k*_β_/*k*_−__β_) [15], it may be varied by 4 orders of magnitude (*k*_−__β_ = 2 × 10^−2^ s^−1^ and *k*_β_ between 1.8 × 10^3^ and 1.8 × 10^7^ L·mol^−1^·s^−1^) or remained constant but changing simultaneously the magnitude of *k*_β_ and *k_−_*_β_ (*k*_β_ between 1.8 × 10^3^ and 1.8 × 10^5^ L·mol^−1^·s^−1^ and *k*_−__β_ between 2 × 10^−3^ and 2 × 10^−1^ s^−1^). Unfortunately, no couple of *k*_β_ and *k*_-β_ other than the values reported in Table 4 allows a better description of the experimental data. The evolution of *Đ* with reaction time matches well with theoretical values for the simulation, but the experimental values of *Đ* are below the theoretical line; i.e., the number-average molecular weights are higher than the theoretical values. This could be due to the fact that PREDICI^®^ software takes into account all species, including very short (dead) oligomers, while SEC analyses do not consider all species (due to the position of the low-molecular weight integration limit and differences in refractive index), which would also lead to values of *M*_n_ above the theoretical line [27]. Nevertheless, a good control of the molecular weight and its distribution were obtained for CPDB-mediated RAFT polymerization of TBDMSMA with *Đ* values lower than 1.2 at high monomer conversion.

### 3.3. Effect of Initiator Concentration

The effect of the initial AIBN concentration in the CPDB-mediated polymerization of TBDMSMA was carried out at 70 °C and with three concentrations of AIBN being 1.5 × 10^−3^, 3.0 × 10^−3^, and 6.0 × 10^−3^ mol·L^−1^. The monomer and CPDB concentrations were fixed at 1.5 mol·L^−1^ and 3.0 × 10^−2^ mol·L^−1^, respectively. For simulations, all the kinetic parameters were set identical to the optimal parameters reported above. 

**Figure 7:**
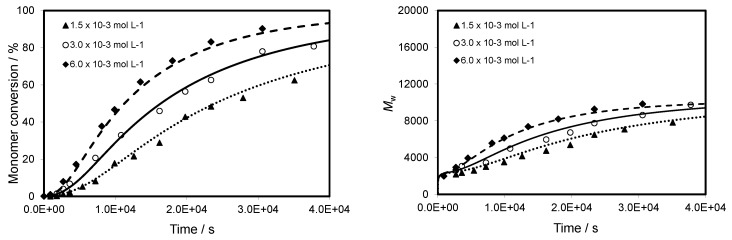
Evolution of monomer conversion and *M*_w_ versus reaction time for CPDB-mediated polymerization of TBDMSMA in toluene at 70 °C with initial concentration of AIBN ranging from 1.5 × 10^−3^ to 6.0 × 10^−3^ mol·L^−1^. The graph compares predicted values (lines) with experimental data (points).

Figure 7 shows a good agreement between the experimental and simulated data with all the three initiator concentrations. An increase in the initiator concentration leads to an increase in the rate of polymerization and in the maximal value of monomer conversion. This behaviour is directly due to an increase in the propagating radical concentration in the polymerization mixture. The use of a very low concentration of AIBN in order to limit the termination reactions can lead to lower monomer conversions. A CTA to AIBN ratio equal to 5 leads to a monomer conversion close to 100 %, whereas only conversions of 90% and 70% are obtained for a CTA to AIBN ratio equal to 10 and 20, respectively.

**Figure 8:**
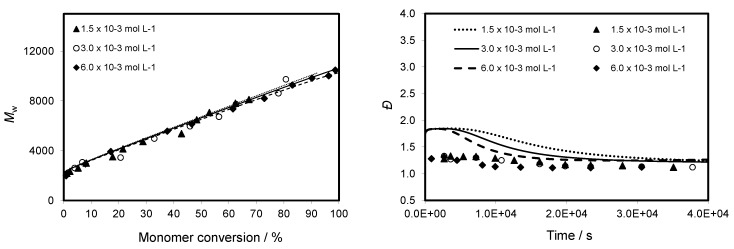
Evolution of *M*_w_ and *Đ* versus monomer conversion for CPDB-mediated polymerization of TBDMSMA in toluene at 70 °C with initial concentration of AIBN ranging from 1.5 × 10^−3^ to 6.0 × 10^−3^ mol·L^−1^. The graph compares predicted values (lines) with experimental data (points).

Figure 7 and Figure 8 show that the evolution of molecular weight with reaction time is faster with increasing AIBN concentration but the evolution of molecular weight with monomer conversion is similar for all the studied AIBN concentrations. This indicates that there is either a minor effect or no effect on the molecular weight with respect to the range of AIBN concentration used. In addition, the *Đ* value decreases during the polymerization and is close to 1.15 at high conversion. A better result is attributed to the CTA to AIBN ratio equal to 5 because we obtained at a given time a higher conversion with a control of the molecular weight and a narrow molecular weight distribution.

## 4. Conclusions

The kinetic study of the CPDB-mediated RAFT polymerization of *tert*-butyldimethylsilyl methacrylate has been investigated. Propagation and termination rate coefficients for free radical polymerization of TBDMSMA have been experimentally determined for various temperatures. In the present study we have demonstrated that the PREDICI^®^ model can be a useful tool to describe in detail the CPDB-mediated RAFT polymerization of TBDMSMA. The rate coefficient for the addition reactions to RAFT agent (*k*_β,1_, *k*_β,2_) and to polymeric RAFT agent (*k*_β_), and for the fragmentation reactions of intermediate radicals in pre-equilibrium (*k*_-β,1_, *k*_-β,2_) and in the main equilibrium (*k*_-β_) have been estimated by fitting the experimental data. It was revealed that a short inhibition period should be induced by slow fragmentation of the intermediate RAFT radicals in the pre-equilibrium. The relatively low *k*_tr_ (i.e., low *C*_tr_) value led to a deviation of the molecular weight at zero conversion. Nevertheless, a good control of the molecular weight and its distribution have been obtained thank to high equilibrium constant of the main equilibrium (*K*_eq_ = *k*_β_/*k*_−__β_). This study also contributes to the development of kinetic databases for free radical and RAFT polymerizations of methacrylate monomers.

## Figures and Tables

**Table 1:** Data from the RAFT polymerizations of TBDMSMA in toluene solution.

Entry	Temperature (°C)	[TBDMSMA] (mol·L^−1^)	[CPDB] (mol·L^−1^)	[AIBN] (mol·L^−1^)	Monomer conversion (%)	*M*_n_ (g·mol^−1^)	*Đ* (*M*_w_/*M*_n_)
1	60	1.5	-	6.0 × 10^−3^	13.9	-	-
2	70	1.5	-	6.0 × 10^−3^	13.9	-	-
3	80	1.5	-	6.0 × 10^−3^	15.2	-	-
4	70	1.5	1.0 × 10^−2^	6.0 × 10^−3^	97.1	24,300	1.10
5	70	1.5	1.5 × 10^−2^	6.0 × 10^−3^	96.9	17,700	1.12
6	70	1.5	3.0 × 10^−2^	6.0 × 10^−3^	99.3	9600	1.11
7	70	1.5	6.0 × 10^−2^	6.0 × 10^−3^	97.5	5400	1.12
8	70	1.5	3.0 × 10^−2^	3.0 × 10^−3^	91.0	9840	1.10
9	70	1.5	3.0 × 10^−2^	1.5 × 10^−3^	71.2	7950	1.10

**Table 2:** *k*_p_ values obtained from PLP-SEC investigations of free-radical polymerization of TBDMSMA in toluene for different temperatures.

Entry	Temperature (°C)	[TBDMSMA] (mol·L^−1^)	*t*_f_ (s)	*DP*	*k*_p_ (L·mol^−1^·s^−1^)	ln*k*_p_
10	50.1	1.502	0.05	49.8	663	6.50
11	50.1	1.502	0.05	47.6	653	6.48
12	60.2	1.502	0.05	58.5	779	6.66
13	60.1	1.502	0.05	58.5	779	6.66
14	70.0	1.502	0.05	75.4	1004	6.91
15	70.7	1.502	0.05	77.1	1027	6.93
16	79.9	1.502	0.05	94.9	1264	7.14
17	80.0	1.502	0.05	86.5	1152	7.05
18	80.4	1.502	0.05	80.8	1075	6.98

*t*_f_: time between pulses; *DP*: degree of polymerization of polymer formed; the *k*_p_ values for TBDMSMA polymerization were determined from the second POI.

**Table 3:** *k*_t_ values for free-radical polymerization of TBDMSMA in toluene at different temperatures.

Temperature (°C)	*k*_t_ (L·mol^−1^·s^−1^)
60	1.49 × 10^7^
70	1.68 × 10^7^
80	1.90 × 10^7^

**Table 4:** Set of fixed input parameters for the PREDICI^®^ simulations of CPDB-mediated polymerization of TBDMSMA in toluene at 70 °C.

Parameter	Value
*k*_d,eff_ (s^−1^)	2.40 × 10^−5^
*k*_i_ (L·mol^−1^·s^−1^)	4.86 × 10^3^
*k*_p_ (L·mol^−1^·s^−1^)	9.72 × 10^2^
*k*_p, rein_ (L·mol^−1^·s^−1^)	4.86 × 10^3^
*k*_t_ (L·mol^−1^·s^−1^)	1.68 × 10^7^
α	1
$c_{\mathrm{monomer}}^{0}$(mol·L^−1^)	1.5
$c_{\mathrm{CTA}}^{0}$(mol·L^−1^)	1.0 × 10^−2^; 1.5 × 10^−2^; 3.0 × 10^−2^; 6.0 × 10^−2^
$c_{\mathrm{AIBN}}^{0}$(mol·L^−1^)	6.0 × 10^−3^

**Table 5:** Final estimated values of rate coefficients for CPDB-mediated polymerization of TBDMSMA in toluene at 70 °C.

Parameter	Value
*k*_β_ (s^−1^)	1.80 × 10^4^
*k*_β,1_ (s^−1^)	1.80 × 10^4^
*k*_β,2_ (s^−1^)	1.80 × 10^4^
*k*_-β_ (s^−1^)	2.0 × 10^−2^
*k*_-β,1_ (s^−1^)	2.0 × 10^−2^
*k*_-β,2_ (s^−1^)	2.0 × 10^−2^

## Supplementary Materials

The following are available online at http://www.mdpi.com/2073-4360/10/2/224/s1, Scheme S1: Reaction scheme of the RAFT process implemented into the PREDICI simulation program, Figure S1: ^1^H NMR spectra of TBDMSMA and PTBDMSMA, Figure S2: SEC chromatograms of PTBDMSMA, Figure S3: Evolution of *M*_w_ (a) and *Đ* (b) versus monomer conversion for CPDB-mediated polymerization of TBDMSMA in toluene at 70 °C with initial concentration of CPDB ranging from 1.5 × 10^−2^ to 6.0 × 10^−2^ mol·L^−1^.
