# Supplementary material for: RAFT Polymerization of Tert-Butyldimethylsilyl Methacrylate: Kinetic Study and Determination of Rate Coefficients
Source: Polymers (Basel). 2018 Feb 24;10(2):224. doi: 10.3390/polym10020224 (PMC6414831; doi:10.3390/polym10020224)
Supplement: Supplementary file 1 [file polymers-10-00224-s001.pdf]

SUPPLEMENTARY MATERIALS

RAFT polymerization of *tert*-butyldimethylsilyl methacrylate: Kinetic study and determination of rate coefficients

Minh Ngoc Nguyen [1,*], André Margaillan [2], Quang Trung Pham [1] and Christine Bressy [2,*]

[1] Faculty of Chemistry, VNU University of Science, Hanoi, 19 Le Thanh Tong, Hoan Kiem, Hanoi, Viet Nam

[2] Université de Toulon, Laboratoire MAtériaux Polymères-Interfaces-Environment Marin (EA 4323 MAPIEM), CS 60584 -83041 Toulon Cedex 9, France

* Correspondence: M.N. Nguyen (E-mail: nmngoc@hus.edu.vn; Tel : +84-243-825-3503); C. Bressy (E-mail: christine.bressy@univ-tln.fr; Tel : +33-49-414-2580)

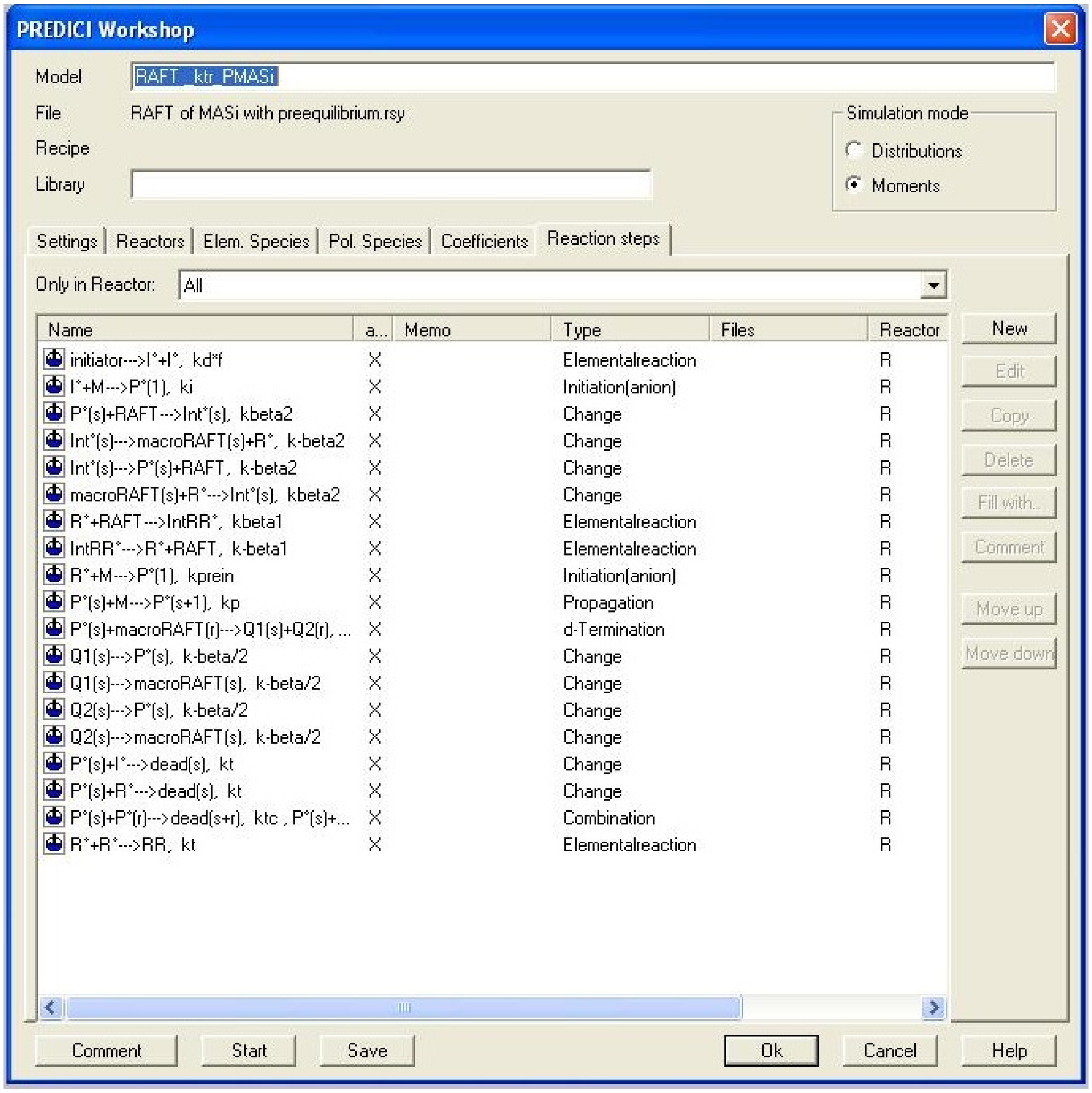

Scheme S1. Reaction scheme of the RAFT process implemented into the PREDICI simulation program.

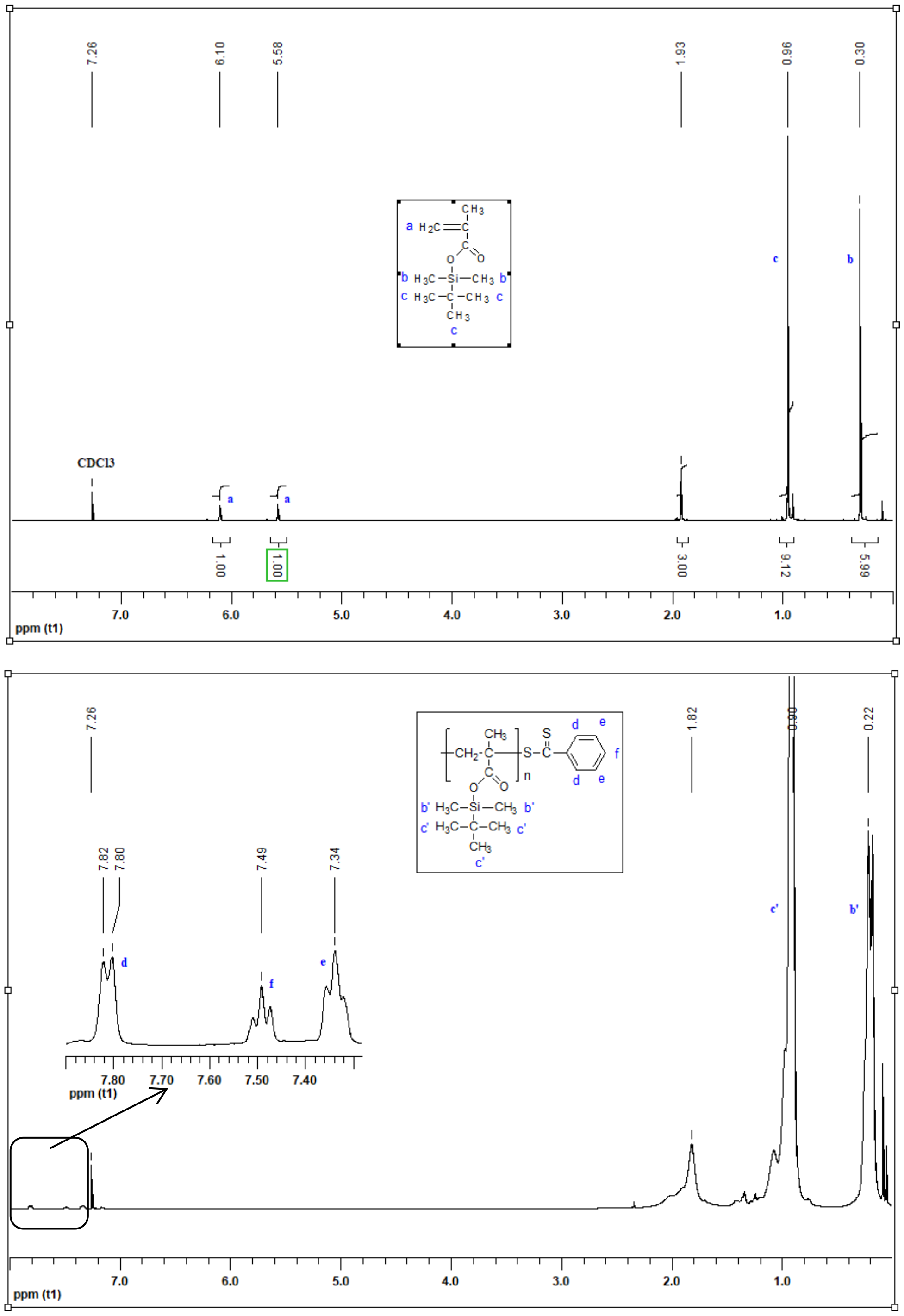

Figure S1. ^{1}H NMR spectra of TBDMSMA and PTBDMSMA.

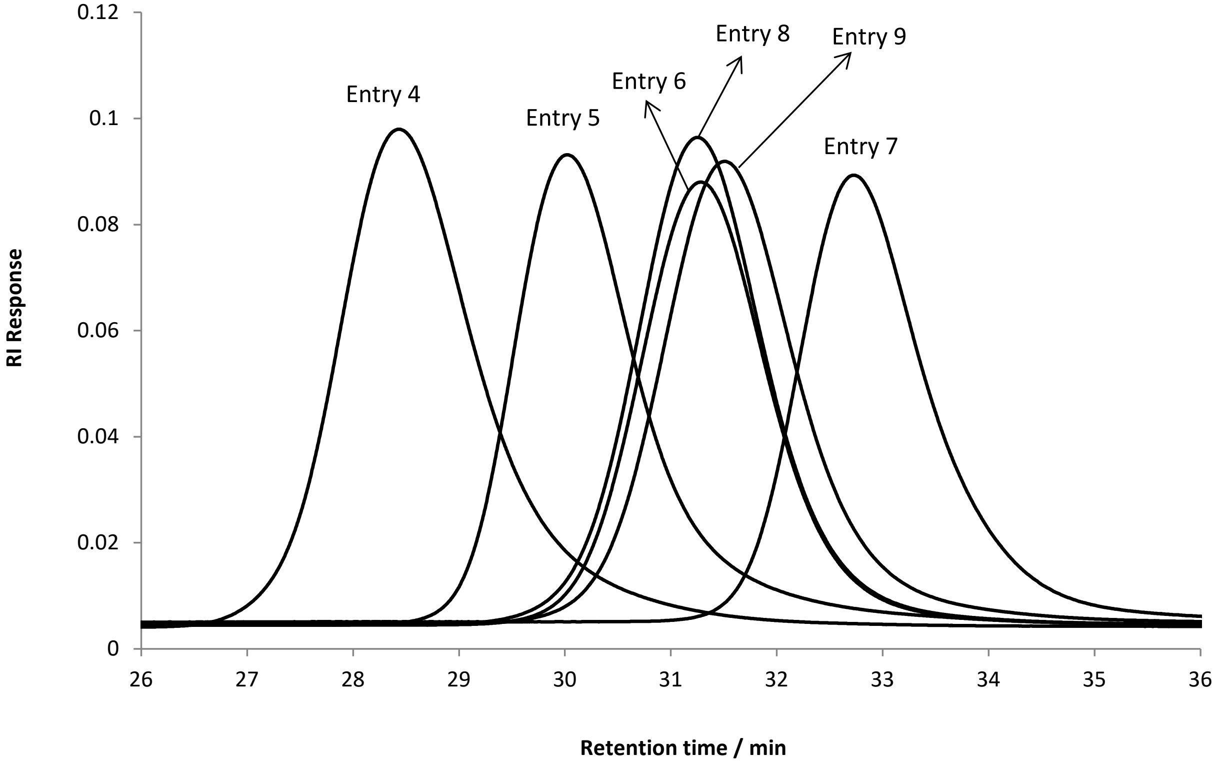

Figure S2. SEC chromatograms of PTBDMSMA.

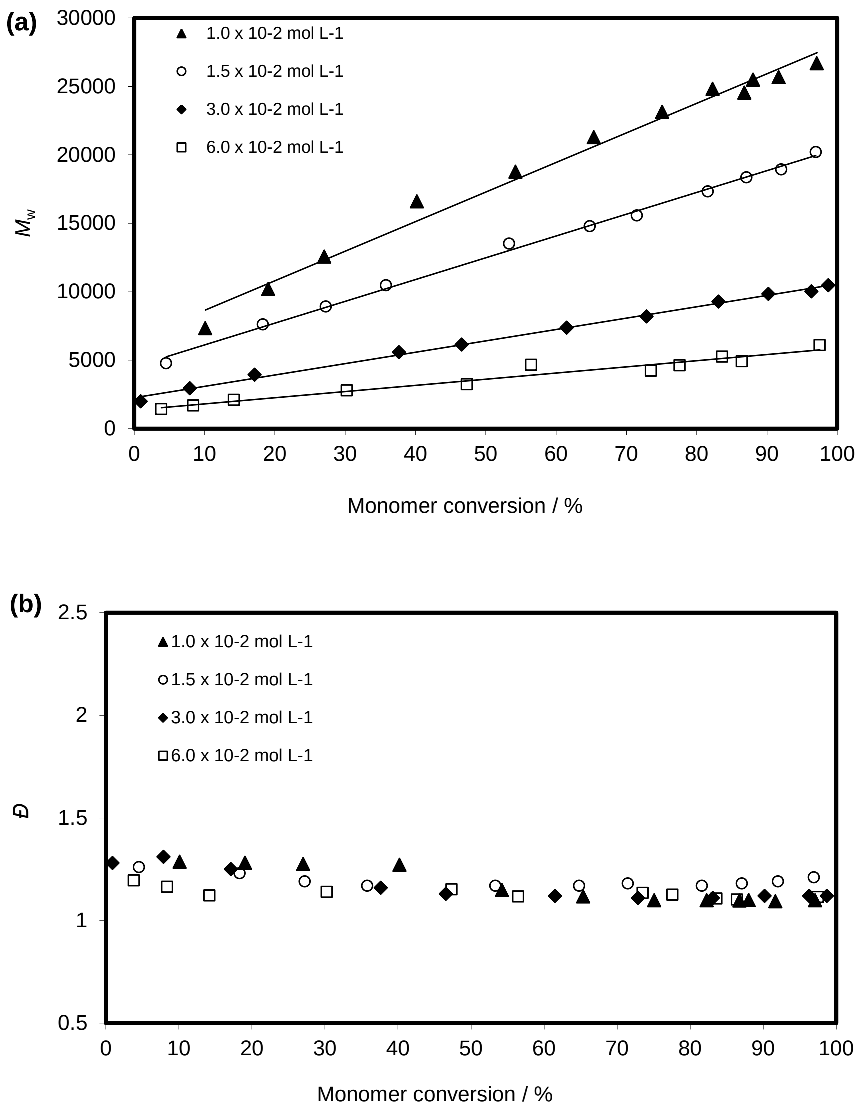

Figure S3. Evolution of M_w (a) and *Đ* (b) versus monomer conversion for CPDB-mediated polymerization of TBDMSMA in toluene at 70 °C with initial concentration of CPDB ranging from 1.5×10^{-2} to 6.0×10^{-2} mol L^{-1}.
